# Exploiting Information and Control Theory for Directing Gene Expression in Cell Populations

**DOI:** 10.3389/fmicb.2022.869509

**Published:** 2022-04-25

**Authors:** Lucas Henrion, Mathéo Delvenne, Fatemeh Bajoul Kakahi, Fabian Moreno-Avitia, Frank Delvigne

**Affiliations:** Microbial Processes and Interactions (MiPI), Terra Research and Teaching Centre, Gembloux Agro-Bio Tech, University of Liège, Gembloux, Belgium

**Keywords:** phenotypic heterogeneity, biological noise, population control, synchronization, cell collective behavior, cell decision-making process

## Abstract

Microbial populations can adapt to adverse environmental conditions either by appropriately sensing and responding to the changes in their surroundings or by stochastically switching to an alternative phenotypic state. Recent data point out that these two strategies can be exhibited by the same cellular system, depending on the amplitude/frequency of the environmental perturbations and on the architecture of the genetic circuits involved in the adaptation process. Accordingly, several mitigation strategies have been designed for the effective control of microbial populations in different contexts, ranging from biomedicine to bioprocess engineering. Technically, such control strategies have been made possible by the advances made at the level of computational and synthetic biology combined with control theory. However, these control strategies have been applied mostly to synthetic gene circuits, impairing the applicability of the approach to natural circuits. In this review, we argue that it is possible to expand these control strategies to any cellular system and gene circuits based on a metric derived from this information theory, i.e., mutual information (MI). Indeed, based on this metric, it should be possible to characterize the natural frequency of any gene circuits and use it for controlling gene circuits within a population of cells.

## Introduction

The parallel advances made at the level of cell culturing procedures [i.e., microfluidics (Grunberger et al., 2014) and cell–machine interfaces (Lugagne and Dunlop, 2019)], as well as the manipulation of gene circuits (Wong and Liao, 2006; Levine et al., 2013; Din et al., 2020), have paved the way for the design of efficient cell population control procedures. It is now possible to act either on cell population (Milias-Argeitis et al., 2016; Sassi et al., 2019; Nguyen et al., 2021) or on individual cells within population (Lugagne et al., 2017; Rullan et al., 2018) for directing gene expression and cellular functions. In this review article, we will focus more precisely on a generic approach that could be used to control gene expression in individual cells among population. A critical aspect that must be taken into account before being able to manipulate gene expression in cell population is related to the inherent noise of cellular systems (Pilpel, 2011). This noise induces cell-to-cell variability in gene expression, and a potential control procedure must be designed by taking into account the inherent functionality exhibited by noise on the cellular system (Levine et al., 2013; Ackermann, 2015). Indeed, it is known that biological noise is a mechanism exploited by cell population in order to increase its fitness in front of fluctuating environmental conditions (Thattai and van Oudenaarden, 2004; Kussell and Leibler, 2005). As an example, in natural ecosystems, microbial populations are often exposed to unpredictable environmental changes such as nutrient starvation, exposure to antibiotics, temperature variations, and many other sources of stress (Ackermann, 2015) that can fluctuate periodically or randomly. Cellular systems have then evolved accordingly by adapting different cellular components in order to accommodate such fluctuations involving different timescales. If environmental conditions change slowly and regularly, a responsive switching strategy leads to increased fitness for the cell population (Kussell and Leibler, 2005). On the other hand, if environmental conditions are fast and erratic, a random switching mechanism, leading to preadapted cells, is more suited for optimizing population fitness. The study of phenotypic diversification mechanisms involved in antibiotic persistence in bacteria has pointed out that cellular systems can take benefit from both stochastic and responsive switching (Kussell et al., 2005). It is clear that, for designing an efficient population control procedure, stochastic switching must be minimized and responsive switching must be favored. Such responsive mechanisms typically involve gene circuits, able to record environmental changes and to respond accordingly. A spectacular realization of the inference of periodic environmental changes by gene circuits is the implementation of circadian (oscillation with a period of ~24 h; Voigt et al., 2016) or ultradian (oscillation with a period < 24 h; Isomura and Kageyama, 2014) rhythms by cellular systems. Many other gene circuit architectures or motifs are known to be able to infer extracellular signals and trigger appropriate biological responses (Perkins and Swain, 2009; Balazsi et al., 2011). Even if we have now access to a classification of the motifs and their possible dynamics (Shoval and Alon, 2010), it is still a challenge to infer the dynamics when several motifs are combined to each other or when the response interferes with many other cellular components. Indeed, in some case, gene circuit architectures can involve overlaps between different stress response pathways, allowing cells to anticipate environmental changes (Tagkopoulos et al., 2008; Freddolino and Tavazoie, 2012). This anticipatory switching arises in ecosystems where different environmental changes exhibit a strongly correlated time profile. As an example, *Escherichia coli* has evolved in order to be able to grow inside and outside a host (i.e., a mammals; Mitchell et al., 2009). When invading the host, *E. coli* is exposed to heat shock where temperature increases from 20 to 37°C. This heat shock is then followed by oxygen limitation as bacteria are reaching the gastrointestinal tract. The gene circuits involved in heat shock response and oxygen limitation have been found to share common inputs and outputs in *E. coli*, elevation of heat leading to the adaptation to oxygen limitation in order to anticipate correlated environmental changes.

Given all these elements, it is then difficult to infer the mode of switching, i.e., stochastic, responsive, or anticipatory (or a combination of them) based on the gene circuit architecture. Accordingly, we propose in this work, a generalizable approach aiming at stimulating the responsive component of switching for directing gene expression in cell population. Such approach could be made possible through the use of a universal metrics aiming at quantifying the information transfer efficiency in cells and leading to the design of robust cell–machine interfaces.

## Using Information Theory for Determining the Optimal Stimulation Frequency Leading to Coordinated Gene Expression in Cell Population

Cells are intrinsically programmed in order to react to external stimuli and to adapt appropriately by switching to different phenotypic states (Acar et al., 2008; Schreiber and Ackermann, 2020). It is then unrealistic to try to keep these cells into a specific phenotypic state, even if this would be a nice outcome for several applications, such as the optimization of cell factories for bioprocessing (Binder et al., 2017). Indeed, these phenotypic states are linked to specific environmental states through selection pressure and the resulting fitness advantage, environmental condition being under constant evolution (Thattai and van Oudenaarden, 2004). A more realistic alternative is to control cell switching itself, which is now technically feasible through the use of cell–machine interfaces (Delvigne et al., 2017; Sassi et al., 2019; Nguyen et al., 2021). In order to make this control strategy successful, two specific aspects must be taken into account, i.e., the efficiency in information transmission through the targeted gene circuits and the timing at which cells commit to phenotypic switching. These two aspects will be illustrated through a case study recently addressed, i.e., the synchronization for the activation of the gene circuit responsible for the induction of the arabinose operon in *E. coli* (Nguyen et al., 2021). The relevance of this case study is also justified by the fact that the arabinose operon has been long used as a biological case study for the characterization of the functionality of biological noise in cell population (Megerle et al., 2008) and also by the fact that the genes belonging to the arabinose operon are widely used for synthetic biology applications and notably for the synchronization of cell response (Stricker et al., 2008; Mondragón-Palomino et al., 2011). Finally, the arabinose operon is known to exhibit strong cell-to-cell variability both in the timing for activation (Megerle et al., 2008; Nikolic et al., 2017) and also the level of expression of the corresponding genes (Sagmeister et al., 2014), suggesting that the underlying cell switching mechanisms involves a mix of responsive and stochastic components. This make this system very interesting to be considered for possible coordination at the population level.

**Figure 1:**
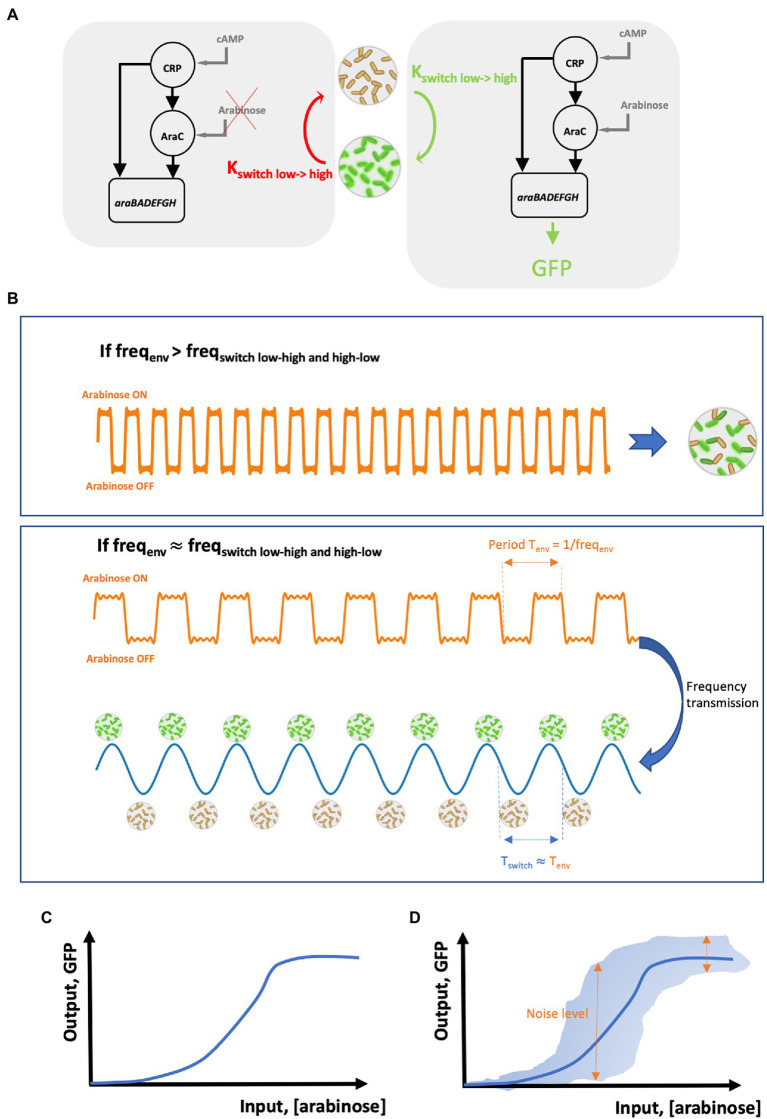
**(A)** Scheme of the feedforward loop motif involved in the regulation of the arabinose operon. On the left, arabinose is not available and the AraC branch cannot be induced. Accordingly, cell switching does not take place and, eventually, previously induced cells are relaxed back to the uninduced (low) state at a rate K_switch low->high_. On the right, arabinose is available and the AraC branch, together with the cAMP-CRP branch, is activated leading to the induction of the genes *araBADEFGH* involved in arabinose metabolism. Under these conditions, cells from the low-state switch actively to the high state at a rate K_switch high->low_. **(B)** Proper coordination/synchronization of gene expression can be achieved based on periodic stimulations (or environmental fluctuations) made at a specific frequency freq_env_. If freq_env_ is too high by comparison with the frequency for cell switching freq_switch_, then cells are not coordinated and exhibit strong variability in gene expression. However, when freq_env_ is set close to freq_switch_, coordination in gene expression is possible leading to synchronized gene expression. **(C)** Typical shape of a Hill relationship between an input (here the concentration of arabinose in the medium) and its resulting output (here, detected based on the synthesis of GFP based on a P*_araBAD_*::GFP transcriptional reporter). **(D)** Impact of biological noise (represented by double arrows) on the probability for delivering an output based on a given input.

The first step in the cell-to-cell coordination for the activation of the arabinose operon is to know the possible effector for the underlying gene circuit. The activation of the arabinose operon is under the control of a feedforward loop (Mangan et al., 2003; Figure 1A) combining the glucose depletion signal (through the accumulation of cAMP inside cells) and the presence of arabinose (through the activation of the transcription factor AraC). Under glucose-limiting conditions, it is then possible to activate or deactivate this gene circuit based on arabinose pulsing (Nguyen et al., 2021; Figure 1A). At this stage, the first drawback exhibited by biological noise can be observed. Indeed, upon arabinose pulsing, cells will commit to the activation of the feedforward circuit leading to the synthesis of the different proteins involved in arabinose assimilation. However, due to biological noise, timing in commitment will exhibit cell-to-cell heterogeneity (Yurkovsky and Nachman, 2013; Ghusinga et al., 2017). Timing in cellular commitment to alternative phenotypes depends on the accumulation of regulatory proteins at the single cell level. Transcription and translation processes in individual cells are prone to biological noise (Thattai and van Oudenaarden, 2001; Swain et al., 2002). These processes can be simulated based on the resolution of the chemical master equation or, more practically, based on the Gillespie algorithm (Thattai and van Oudenaarden, 2001). These simulations have been shown to lead to very realistic pictures for mRNA and protein synthesis in individual cells (Balazsi et al., 2011) and pointed out that these processes follow Poisson statistics. Accordingly, the transition of cells between two adjacent phenotypic states (for example, the two states, GFP negative and GFP positive, drawn in Figure 1A) can also be represented by a Poisson process. One key property of the Poisson process is that the timing between two consecutive events (e.g., the time between the synthesis of two mRNAs from the same DNA sequence in a single cell) follows an exponential distribution. Based on this statement, the residence time distribution of cells in a given phenotypic state can be represented by an exponential distribution (Norman et al., 2015). It is thus very critical to take into account this residence time distribution for coordinating cell switching at the population level. One way to overcome this use is to rely on the use of a cell–machine interface allowing the on-line monitoring of the switching process at the level of individual cells in the population and to react accordingly. This principle has been notably adopted for developing the segregostat (Sassi et al., 2019; Nguyen et al., 2021). This system is based on the use of on-line flow cytometry for recoding the cell switching rate and to trigger environmental switching accordingly. The fact that the frequency of environmental perturbation must be set based on the phenotypic switching frequency has been previously deduced from numerical simulation (Thattai and van Oudenaarden, 2004). In a similar way, another study has pointed out that the control of gene circuits is dramatically reduced above a critical stimulation frequency (Tan et al., 2007). Under these conditions, the frequency of the extracellular signal is effectively transmitted, leading to a cell population with synchronized gene expression (Figure 1B). Effective entrainment of cell population can be assessed based on the oscillatory gene expression profile exhibiting a frequency close to the one of the input stimulations. Such oscillations were experimentally observed during segregostat experiments carried out for controlling the activation of the arabinose operon (Nguyen et al., 2021). It is also important to point out that in this case, square waves are used as stimulatory input. This strategy is also called pulse width modulation (PWM; Davidson et al., 2013; Purvis and Lahav, 2013). We will see in the next section that this strategy has been used several times for controlling different cellular systems (listed in Table 1). In the present case, population oscillates according to a frequency corresponding to the one of the input square waves. This is represented in Figure 1B based on the period T_env_ (T_env_ = 1/frequency) of the input square wave stimulation, which is transmitted to the population and lead to oscillation in gene expression with the same period T_switch_ = T_env_.

**Table 1:** Range of environmental stimuli used for controlling gene expression in cell population and range of periodic signal associated with these environmental perturbations.

Approximated period T for the input stimulation^A^	Type of perturbation^B^	Controlled trait	Organism	Culture system	Single-cell analysis tool	References
83 min^*^	Pulse of phosphate in phosphate-poor medium	Cell cycle	*E. coli*	Bioreactor 240 ml	None	Goodwin, 1969
98 min^*^	Pulses of methionine to induce (*MET3*pr-*CLN2*)	Cell cycle	*S. cerevisiae*	Microfluidics	Microscopy	Charvin et al., 2009
110 min^*^ 150 min^*^	Nutrient availability, i.e., poor-rich	Cell cycle	*S. cerevisiae*	Microfluidics	Microscopy	Tian et al., 2012
16 min 45 min	Red-far red pulses of light	*gal1*-responsive genes through optogenetic	*S. cerevisiae*	Microplates with 96 wells	Flow cytometry	Milias-Argeitis et al., 2011, 2016
28 min 88 min	Pulses of sorbitol-enriched medium in normal medium	Osmostress (value of 1,500 a.u.)	*S. cerevisiae*	Microfluidics	Microscopy	Uhlendorf et al., 2012
142 min	Glucose–galactose	p*gal1*-GFP	*S. cerevisiae*	Microfluidics	Microscopy	Fiore et al., 2016
~990 min	Tetracycline	Tetracycline-inducible system p(CMV-TET)-d2EYFP	Mammalian (CHO)	Microfluidics	Microscopy	Fracassi et al., 2016
120 min	Green light intensity (in relation with red light intensity)	CcaS/CcaR gene expression system at a varying level of expression	*E. coli*	Bioreactor 20 ml	Automated flow cytometry	Milias-Argeitis et al., 2016
6 min	Computed open-loop green–red light	ccaSR-based system	*E. coli*	Microfluidics	Microscopy	Chait et al., 2017
150 min 210 min (depending on the amplitude/concentration)	IPTG—aTc	Maintain a toggle switch at an unstable intermediary level	*E. coli*	Microfluidics	Microscopy	Lugagne et al., 2017
10 min	High and low red intensity blue light	Transcription	*S. cerevisiae*	Microfluidics	Microscopy	Rullan et al., 2018
For GFP: −5 s off/80s on −8 s off/80 s on −11 s off/80 s on For isobutanol and butanol: −65 s off and 15 s on	Blue light	-GFP expression -production of isobutanol and 2-methyl-1-butanol	*S. cerevisiae*	For GFP: Microplates 24 wells For isobutanol and butanol: Bioreactor 0.5 L	None	Zhao et al., 2018
5–60 min	Pulses of galactose to stabilize synecluine concentration at different values	a-synuclein formation	*S. cerevisiae*	Microfluidics	Microscopy	Perrino et al., 2019
90 min 105 min 180 min	Methionine concentration in cultivation medium	Yeast cell cycle coordination	*S. cerevisiae*	Microfluidics	Microscopy	Perrino et al., 2021
600 min	Arabinose pulses	Arabinose operon induction	*E. coli*	Bioreactor 1 L (continuous mode)	Online flow-cytometry	Nguyen et al., 2021

A This column indicates the range of periodic signal associated with these environmental perturbations. In most of the case, the input signal can be approximated by a square wave of period T and frequency 1/T (see Figure 1B for more details).

B This column indicates the range of environmental stimuli used for controlling gene expression.

* These stimulation periods have been determined without monitoring and feedback control (open-loop control).

All these observations point out that cells are able to deduce changes in their surroundings based on diverse sensory mechanisms. We do not want here to discuss about the biological diversity of these mechanisms, but rather to quantify the efficiency at which a cell is able to infer extracellular perturbation. A universal way to quantify information transmission through biochemical network can be derived from Shannon theory or information theory (Cheong et al., 2011). In order to be able to understand the importance of information theory in biochemical signal processing, it is important to introduce the concept of input–output (i/o) or dose–response relationship. For many gene circuits, this i/o relationship can be represented by a sigmoidal curve (Figure 1C), also called Hill equation (Levchenko and Nemenman, 2014). For example, Hill equation can be used to infer the response of the feedforward loop involved in the regulation of the arabinose operon (Mangan and Alon, 2003). This i/o correlation tells us what will be the output of the gene circuit according to a given input. However, we have seen that cell switching mechanism involves a random component in addition to the responsive one. This random component can be represented by the error bars on the i/o correlation (Figure 1D). Accordingly, one input can drive different output trajectories, leading to cell-to-cell heterogeneity. It can be seen that some input leads to a very heterogeneous response, making cell unable to properly infer the state of the environment. This is exactly where information theory can be useful, i.e., by providing a metric for quantifying the amount of information transmitted by the gene network for some specific input environmental conditions. This metric, mutual information (MI), corresponds to the logarithm of the number of distinct, input-dependent, states that can be reached by cells (Levchenko and Nemenman, 2014) and is quantified in bits. For example, a gene network exhibiting a MI of one bit means that only two physiologically distinct states can be resolved by cells based on the input conditions. Generally speaking, most of the gene circuits are corrupted by noise and can carry only a limited amount of information, and most of the studies carried out so far in this area have pointed out that MI equals to only 1–2 bits for different gene networks and model organisms (Mehta et al., 2009; Perkins and Swain, 2009; Cheong et al., 2011; Hansen and O’Shea, 2015; Sarkar et al., 2020). This leads to the conclusion that only these states have to be targeted when designing a cell population control strategy.

The next section will be dedicated to the description of some realization in the field of cell population control (also termed cybergenetics), pointing out that the above-mentioned methodology could help at this level by providing a general framework aiming at developing further cell population control procedures.

## The Contribution of Control Theory and the Nascent Field of Cybergenetics

The fact that cell population can be controlled based on pulsatile inputs has been reported a long time ago. Indeed, long before the advent of single-cell technologies, Goodwin (1969) observed that it was possible to synchronize cell cycle in *E. coli* cells by periodically pulsing phosphate in a phosphate-limited chemostat. This pioneering work has led to the establishment of a robust modeling framework for the understanding of the impact of external conditions on the synchronization of cell cycle for many types of organisms (Ruoff et al., 2001; Gonze and Abou-Jaoudé, 2013; Gonze and Ruoff, 2021). However, these studies have been carried out based on an open-loop control approach and the application of regular pulses with varying frequencies and amplitudes. More recently, the application of control theory to the manipulation of cellular systems, i.e., cybergenetics, has set the ground for a more rational design of cell population control procedures (Milias-Argeitis et al., 2016; Banderas et al., 2020). Cybergenetics is an entirely new and exciting field of research at the interface between control engineering and synthetic biology, and emerged with the recent advances made in genetic engineering combined with the works initially derived from cybernetics (Wiener, 1961). A distinction can be made between “internal cybergenetics” (also called *in vivo* and involving genetic controllers directly embedded in cells) and “external cybergenetics” (also called *in silico* controllers; Lugagne et al., 2017; Lugagne and Dunlop, 2019; Carrasco-López et al., 2020; Pedone et al., 2021). In the context of this review, we will be focused more on the latter technology, since it involves cell–machine interface and pulsatile inputs used as actuators.

Remarkably, although different systems have been used (e.g., various model organisms, type of gene circuits to be controlled, and single cell techniques), all the data accumulated point out that it is possible to effectively control gene circuits at the level of individual cells by applying external periodic signals (Table 1). Evidences have been provided suggesting that pulses of inducers tend to decrease noise in biochemical network, leading to synchronized gene expression (Uhlendorf et al., 2012; Benzinger and Khammash, 2018). This effect can be explained based on the dose–response relationship (Figure 1D) where input concentrations at the extremities of the dynamic range lead to a homogenous response at the population level. In contrast, input concentrations at the center of the dynamic range produce a heterogeneous population. This strategy, known as PWM, seems to be generalizable for the effective control of diverse gene circuits in diverse cellular systems. Most of the experiments involving the control of gene expression in cellular systems have been performed in microfluidic devices (Table 1). This type of cultivation device allows the acquisition of single cell data with a high spatiotemporal resolution, but with a low experimental throughput due to the time and computational power required for image analysis (Dusny and Schmid, 2014) and with possible technical biases by comparison with conventional cultivation devices (Dusny et al., 2015; Westerwalbesloh et al., 2017). Nonetheless, there is a growing interest in using standard cultivation devices (e.g., flasks, bioreactors, etc.) for studying and controlling cell populations (Zhao et al., 2021). In this case, single-cell analyses can be performed based on automated flow cytometry, leading to the rapid accumulation of data at the population level. In this context, the use of cell–machine interface relying on flow cytometry can lead to the automated determination systematic determination of the optimal stimulation frequency for the effective synchronization of gene expression at the population level (Nguyen et al., 2021).

## Perspective: Exploiting Intrinsic Frequency of Gene Circuits

Taken altogether, the elements assembled in the previous sections point out that a lot of different gene circuits architectures can exhibit periodic behavior [and not only the motifs reported to behave as natural oscillators, such as the repressilator (Elowitz and Leibler, 2000) or the oscillator motif (Stricker et al., 2008; Mondragón-Palomino et al., 2011)] if stimulated at the appropriate frequencies (Tan et al., 2007). Development made in information theory and in cybergenetics provides the computational framework and the experimental tools in order to generalize this concept to many biological systems. Impressive achievements can be expected from these field of research such as the control of complex cell regulatory program (e.g., control of cell cycle program; Perrino et al., 2021) and the control of microbial communities composition (Fiore et al., 2017; Liao et al., 2019), with applications in various field from bioproduction (Briat and Khammash, 2018; Zhao et al., 2021) to biomedicine (Davidson et al., 2013; Din et al., 2020).

## Author Contributions

FD drafted the manuscript, designed Figure 1, and wrote section “Using information theory for determining the optimal stimulation frequency leading to coordinated gene expression in cell population.” FB and FM-A wrote section “Introduction.” LH and MD wrote sections “The contribution of control theory and the nascent field of cybergenetics” and “Perspective: exploiting intrinsic frequency of gene circuits” and designed Table 1. FB, FM-A, LH, and MD revised the final version of the manuscript. All authors contributed to the article and approved the submitted version.

## Funding

LH is supported by the FRS-FNRS (Fond National pour la Recherche Scientifique, Belgium) through a FRIA PhD grant. MD is supported by a FNRS PhD grant in the context of an Era-Net Aquatic Pollutant project (ARENA).

## Conflict of Interest

The authors declare that the research was conducted in the absence of any commercial or financial relationships that could be construed as a potential conflict of interest.

## Publisher’s Note

All claims expressed in this article are solely those of the authors and do not necessarily represent those of their affiliated organizations, or those of the publisher, the editors and the reviewers. Any product that may be evaluated in this article, or claim that may be made by its manufacturer, is not guaranteed or endorsed by the publisher.
